# Baseline medication load and long-term outcomes in COVID-19-hospitalized patients: results of the AUTCOVSTUDY

**DOI:** 10.3389/fpubh.2025.1565677

**Published:** 2025-06-11

**Authors:** Alexandra Graf, Berthold Reichardt, Christine Wagenlechner, Pavla Krotka, Denise Traxler-Weidenauer, Michael Mildner, Julia Mascherbauer, Clemens Aigner, Johann Auer, Ralph Wendt, Hendrik J. Ankersmit

**Affiliations:** ^1^Center for Medical Data Science, Medical University of Vienna, Vienna, Austria; ^2^Austrian Social Health Insurance Fund, Eisenstadt, Austria; ^3^Clinic of Thoracic Surgery, Medical University of Vienna, Vienna, Austria; ^4^Laboratory for Cardiac and Thoracic Diagnosis, Regeneration and Applied Immunology, Vienna, Austria; ^5^Department of Oral and Maxillofacial Surgery, Medical University of Vienna, Vienna, Austria; ^6^Clinic of Dermatology, Medical University of Vienna, Vienna, Austria; ^7^Department of Internal Medicine 3, University Hospital St. Poelten, St. Poelten, Austria; ^8^Department of Internal Medicine I with Cardiology and Intensive Care, St. Josef Hospital Braunau, Braunau am Inn, Austria; ^9^Department of Nephrology, Hospital St. Georg Leipzig, Leipzig, Germany

**Keywords:** COVID-19 hospitalization, all-cause mortality, polypharmacy, baseline medication load, readmission, registry-based observational study

## Abstract

**Introduction:**

Limited data are available on long-term morbidity and mortality after hospitalization for coronavirus disease 2019 (COVID-19). In this registry-based study, we investigated long-term mortality and morbidity following hospitalization for COVID-19 and examined associations with baseline medication load.

**Methods:**

Data were provided by the Austrian Health Insurance Funds on hospitalized COVID-19 patients in 2020 and matched controls. The primary outcome was mortality. Secondary outcomes included mortality conditional on survival of initial COVID-19 hospitalization and re-hospitalization.

**Results:**

The median follow-up was 600 days. A total of 22,571 patients aged >18 were hospitalized in Austria in 2020 due to COVID-19. The risk of mortality was significantly higher with polypharmacy. With the exception of the youngest age group (19–40 years), patients receiving antiepileptics, antipsychotics, or iron supplements, erythropoiesis-stimulating agents, vitamin B12, or folic acid in the year before hospitalization were significantly associated with a higher risk of mortality (all *p* < 0.001). For patients with prescribed non-steroidal anti-inflammatory drugs (NSAIDs) and other anti-inflammatory drugs, significantly increased survival was observed (all *p*-values <0.001). Patients had a higher medication load than the control population. Long-term mortality and the risk of re-hospitalization for any reason were also significantly higher among patients.

**Discussion:**

Antipsychotics and antidepressants appear to be underrecognized in identifying patients at risk for severe outcomes after hospitalization for COVID-19.

## 1 Introduction

The outbreak of Coronavirus Disease 2019 (COVID-19) marked the beginning of a global pandemic and has been associated with substantial morbidity and mortality. Age and sex are well-established risk factors for severe outcomes (1–5). Numerous reports have discussed how the presence of comorbidities may increase the risk of COVID-19-related deaths (1, 6–9). In addition, an increased risk of readmission and mortality after hospital discharge has been observed up to 1 year after hospitalization for COVID-19 (10–13). Patients who require hospitalization for COVID-19 have a greater comorbidity burden and are expected to have worse short-term outcomes (12). Differences in the prevalence of drug use and polypharmacy regimens have been observed when compared to the general population (14). Studies have investigated the potential associations between polypharmacy and increased morbidity and mortality among patients with COVID-19 (15, 16). Visser et al. (17) investigated the impact of polypharmacy on COVID-19-related mortality in nursing home residents and found a significant positive association between the total number of medications and 30-day COVID-19-related adjusted mortality. However, published studies focusing on the drug profiles of hospitalized COVID-19 patients and long-term outcomes are still scarce.

In this study, we present data on hospitalized patients with COVID-19 and a matched control group provided by the Austrian Health Insurance Funds, with a median follow-up of 600 days (and a maximum follow-up of 880 days). The primary aim of this study was to analyze the long-term follow-up of patients hospitalized due to COVID-19 in Austria in 2020 to re-evaluate the association between baseline medication load before hospitalization and mortality or readmission after COVID-19 hospitalization, to go beyond the classical risk factors, and to understand how to better identify and phenotype vulnerable populations of respiratory viral pathogens. We also compared the characteristics and outcomes of the hospitalized patients with an age-, sex-, and region-matched control group, presenting a real-world picture.

## 2 Methods

### 2.1 Study design and cohorts

This original research is based on a retrospective, national registry-based study that complied with the Declaration of Helsinki and was approved by the Ethics Committee of Lower Austria (GS1-EK-4/747–2021). Data on the patient and control cohorts were obtained from the Austrian Health Insurance Funds. Approximately 98% of the Austrian population is registered in the public health insurance system. Austria’s healthcare system is national, with broad access to care, is regulated by social insurance law, and is primarily financed by social insurance contributions.

Patients aged >18 years who were hospitalized in Austria with a primary diagnosis of COVID-19 (ICD-10 codes U071, U072, U049) between 1 January 2020 and 31 December 2020 were included in this study. For all patients, age, sex, region, and medication were obtained 1 year before hospitalization until the study ended.

An age-, sex-, and region-matched control group (approximately 10 controls for each patient) consisted of individuals who were not hospitalized due to COVID-19 in the year 2020 and were randomly selected from the population registered with the Austrian Health Insurance Fund, representing the Austrian population. Data on the control group were available from 1 year before the first patient was hospitalized until the study ended.

### 2.2 Study outcomes

The primary outcome was all-cause mortality. The secondary outcome was hospitalization for any reason. For patients, hospitalization was defined as the first readmission after the index COVID-19 hospitalization. For controls, hospitalization was defined as the first hospital admission after the index COVID-19 hospitalization of the matched patient (Supplementary Table S1). For controls, time to death was evaluated from the index COVID-19 hospital admission date of the matched patient.

### 2.3 Definitions and statistical analyses

For each patient, age, sex, region, and Anatomical Therapeutic Chemical (ATC) Classification Codes describing prescribed medications were available from the Austrian Health Insurance Fund. All analyses were performed separately for four age subgroups: 19–40 years, 41–64 years, 65–74 years, and ≥75 years (Supplementary Table S2). ATC codes available 1 year before hospitalization due to COVID-19 were summarized in the medication groups (Supplementary Table S3). Twenty medication groups were used for statistical modeling (MG1 to MG20, see Supplementary Table S3). For controls, a similar medication profile was generated using the drugs prescribed 1 year before the index COVID-19 hospitalization of the matched patient. These 20 medication groups were also used to define polypharmacy. COVID-19 therapies during hospitalization were not included in the definitions. Since the ATC codes were accurately documented in the Austrian Health System, the medication profile was used to describe the health status of a patient or control.

Numbers and percentages were used to summarize categorical variables, while medians and interquartile ranges were used for continuous variables.

To evaluate the associations between polypharmacy and all-cause death, simple Cox regression models were calculated while adjusting for sex, age, half-year, and polypharmacy, with region as a clustering variable. Polypharmacy was defined as the number of medication groups in which a patient received prescribed medication and was categorized into four groups (0–1, 2–5, 6–10, and >11). To evaluate the association between polypharmacy and re-hospitalization due to any reason, competing risk models (with competing risk of death) were calculated, accounting for sex, age, half-year, and polypharmacy with region as a clustering variable. Furthermore, to evaluate the association between several medication groups and all-cause death, a Cox regression model was calculated, accounting for sex, age, half-year, polypharmacy, and the 20 medication groups, with region as a clustering variable. For hospitalization, a competing risk model was calculated using the same covariables, as described in the model for all-cause mortality.

Due to underrepresentation, only 20 of the 32 medication groups were evaluated for the statistical models.

As patients hospitalized due to COVID-19 potentially had more serious comorbidities compared to the general Austrian population, we attempted to account for this imbalance by performing propensity score matching (PSM) for age, sex, region, and medication groups MG1 to MG20. To evaluate the difference between patients and controls in the risk of all-cause death, a Cox regression model was calculated, adjusting for group, sex, age, half-year, polypharmacy, and the 20 medication groups, with region as a clustering variable. For hospitalization due to any reason, a competing risk model was calculated using the same covariables, as described in the model for all-cause death.

Schönfeld residuals were used to evaluate the proportional hazard assumption, and variance inflation factors were used to evaluate multicollinearity. For all models, hazard ratios (HRs) and confidence intervals (CIs) are provided. Due to the large sample size and the large number of covariables investigated (i.e., 25), a *p*-value <0.002 (=0.05/25 applying conservative Bonferroni correction) was considered significant. All analyses were performed using R software, release 4.2.2 (18). A detailed statistical analysis statement, including definitions, can be found in the supplemental material, Section 3.

## 3 Results

### 3.1 Characteristics of the patient and control cohorts

**Figure 1:**
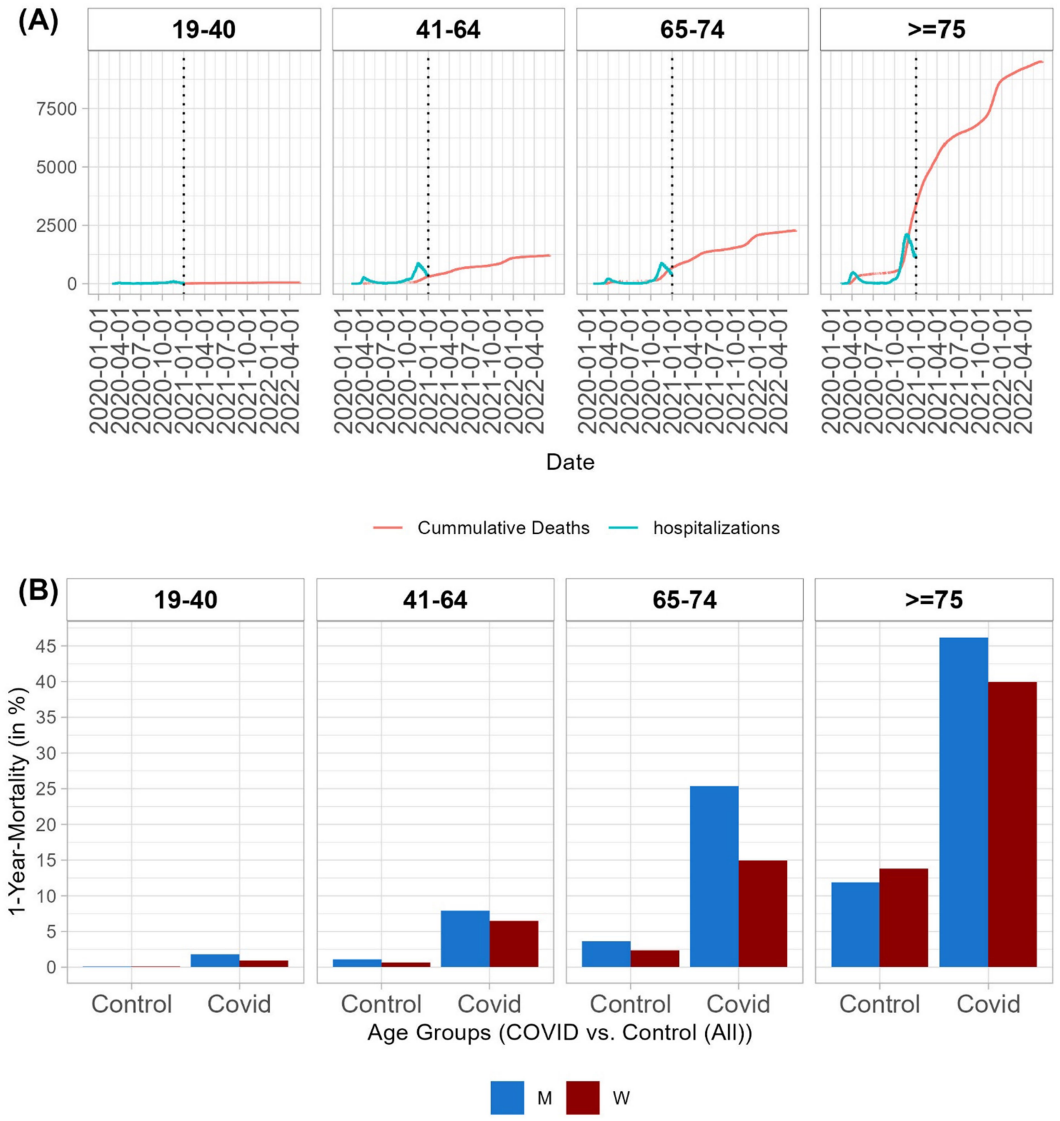
Mortality by age group. **(A)** Timeline of hospitalizations due to COVID-19 in 2020 (hospitalizations per day in blue) and timeline of deaths (cumulative number of deaths in red) of hospitalized patients with COVID-19. **(B)** One-year mortality rates by age group and sex for hospitalized patients due to COVID-19 and controls. For details on mortality rates for patients and controls, along with patients who survived the hospitalization with and without propensity score matching, see Supplementary Tables S8,S9.

The study population included 22,571 COVID-19-hospitalized patients (Table 1 and Figure 1A). The median follow-up time varied from 594 to 615 days across the four age groups. The age-, sex-, and region-matched control groups included 217,295 controls (Table 1).

**Table 1:** Demographic data for hospitalized patients with COVID-19 in 2020 in Austria and corresponding age-, sex-, and region-matched controls.

**All patients**									
		**Age group**							
		19–40		41–64		65–74		≥75	
		*n* = 1,201		*n* = 6,018		*n* = 4,502		*n* = 10,850	
**Parameter**	**Description**	*n*	%	*n*	%	*n*	%	*n*	%
Sex	Male	665	55.37	3,769	62.63	2,644	58.73	4,994	46.03
	Female	536	44.63	2,249	37.37	1,858	41.27	5,856	53.97
Polypharmacy	0	364	30.31	906	15.05	253	5.62	473	4.36
	0 or 1	686	57.12	1929	32.05	619	13.75	1,129	10.41
	2 to 5	454	37.80	2,991	49.70	2,261	50.22	5,106	47.06
	6 to 10	57	4.75	992	16.48	1,400	31.10	4,179	38.52
	≥11	4	0.33	106	1.76	222	4.93	436	4.02
Follow-up time in days	Median (IQR)	615 (590–673)		599 (582–637)		597 (580–615)		594 (577–611)	
Time to death in days	Median (IQR)	47 (11–87)		29 (12–111)		23 (9–131)		16 (7–84)	

Patients surviving hospital stay									
		19–40		41–64		65–74		≥75	
		*n* = 1,192		*n* = 5,727		*n* = 3,854		*n* = 7,674	
		*n*	%	*n*	%	*n*	%	*n*	%
Sex	Male	658	55.20	3,575	62.42	2,177	56.49	3,318	43.24
	Female	534	44.80	2,152	37.58	1,677	43.51	4,356	56.76
Polypharmacy	0	362	30.37	822	15.40	227	5.89	344	4.48
	0 or 1	682	57.21	1,877	32.77	561	14.56	839	10.93
	2 to 5	452	37.92	2,858	49.90	1,985	51.50	3,677	47.92
	6 to 10	54	4.53	903	15.77	1,130	29.32	2,874	37.45
	≥11	4	0.34	89	1.55	178	4.62	284	3.70
Length of hospitalization	Median (IQR)	5 (3–9)		8 (5–13)		11 (6–18)		13 (8–21)	
Follow-up time in days	Median (IQR)	615 (590–673)		599 (582–637)		597 (580–615)		594 (577–611)	
Time to death in days	Median (IQR)	57.5 (47–278)		209 (56–377)		211 (74–389)		160 (49–371)	

Controls									
		19–40		41–64		65–74		≥75	
		*n* = 11,958		*n* = 59,057		*n* = 43,511		*n* = 102,769	
		*n*	%	*n*	%	*n*	%	*n*	%
Sex	Male	6,616	55.32	36,870	62.43	25,353	58.27	47,038	45.77
	Female	5,342	44.67	22,187	37.56	18,158	41.73	55,731	54.22
Polypharmacy	0	9,206	76.99	40,983	69.40	24,535	56.39	42,833	41.68
	0 or 1	10,773	90.09	47,098	79.75	27,877	64.07	48,803	47.49
	2 to 5	1,139	9.53	10,153	17.19	11,322	26.02	32,629	31.75
	6 to 10	45	0.38	1,650	2.79	3,878	8.91	19,285	18.77
	≥11	1	0.01	156	0.26	433	1.00	20,48	1.99
Follow-up time in days	Median (IQR)	614 (590–673)		599 (582–633)		596 (578–613)		591 (574–608)	
Time to death in days	Median (IQR)	158 (62–441)		130 (53–263)		135 (54–297)		138 (47–314)	

Polypharmacy involving more than six medication groups was observed more frequently in the older age groups (65–74 years and ≥75 years). Among these older age groups, 5.62% of patients aged 65–74 years and 4.36% of patients aged ≥75 years, and, among controls, 56.39% of patients aged 65–74 years and 41.68% of patients aged ≥75 years did not receive any medication from the investigated medication groups (Table 1). It should be noted that polypharmacy may be interpreted as an indicator for patients with several underlying diseases and thus potentially identifies risk groups. Patients hospitalized due to COVID-19 are expected to have more comorbidities and, consequently, a higher medication burden than the general population. This pattern was also observed in our Austrian population. Patients received more medications in the year before hospitalization in all investigated medication groups (Table 2 and Supplementary Tables S4–S7) across all age groups compared to controls.

**Table 2:** Percentages of patients hospitalized with COVID-19 and controls who received at least one medication from the investigated medication group 1 year before COVID-19 hospitalization and the corresponding 1-year mortality rates.

	**Age groups**															
	**19–40**				**41–64**				**65–74**	**≥75**
	COVID		Control		COVID		Control		COVID		Control		COVID		Control		Medication group
**%** Yes	**%** 1-year mort	**%** Yes	**%** 1-year mort	**%** Yes	**%** 1-year mort	**%** Yes	**%** 1-year mort	**%** Yes	**%** 1-year mort	**%** Yes	**%** 1-year mort	**%** Yes	**%** 1-year mort	**%** Yes	**%** 1-year mort
MG1: Anticoagulants	6.08	4.11	1.42	1.76	18.79	13.53	4.61	4.45	40.18	26.81	13.05	8.63	56.97	46.59	29.03	23.47
MG2: Antibiotics. antivirals, antiprotozoals, or anthelmintics	46.88	1.60	15.74	0.16	48.31	8.81	15.84	2.02	47.71	22.72	20.19	6.06	46.59	46.96	28.03	22.15
MG3: Insulin and other antidiabetics	3.08	5.41	0.33	0.00	14.69	12.33	2.52	3.36	26.45	26.78	7.29	7.79	21.49	44.13	9.31	23.41
MG4: Heart drugs	1.75	0.00	0.43	1.96	4.54	12.09	1.24	3.67	10.17	26.42	3.93	8.48	16.50	48.27	10.05	23.16
MG5: Antihypertensives, including diuretics and renin-angiotensin-aldosterone system inhibitors	6.33	3.95	0.69	3.66	33.57	11.14	8.66	2.81	60.60	22.84	23.27	5.67	68.89	43.18	39.36	19.62
MG6: Beta-blockers	3.16	5.26	0.27	6.25	14.36	14.24	3.33	4.67	31.05	26.11	10.54	7.63	35.80	45.19	19.52	20.67
MG7: Statins and fibrates, including proprotein convertase subtilisin/kexin type 9 inhibitors and inclisiran	2.25	7.41	0.33	0.00	23.30	9.20	6.57	1.83	42.78	21.7	18.28	4.93	38.55	39.16	24.30	14.98
MG8: Immunosuppressants and immunomodulators	3.00	2.78	0.32	0.00	3.86	9.91	0.70	2.68	4.64	24.40	1.11	5.57	2.15	40.34	1.02	15.39
MG9: Systemic steroids	6.99	4.76	1.16	0.00	12.35	10.90	3.38	4.41	16.79	28.31	6.19	9.17	14.27	43.8	8.37	18.68
MG10: Chemotherapy	0.67	0.00	0.04	20.00	1.83	30.91	0.41	17.77	3.55	41.88	1.34	20.41	3.96	49.53	2.39	28.83
MG11: Iron supplements, erythropoiesis-stimulating agents, vitamin B12, and folic acid	6.16	2.70	1.05	0.00	5.28	22.96	0.91	10.02	9.15	34.95	1.97	17.04	13.00	54.85	5.37	34.64
MG12: Antacids, including antihistamines	10.82	3.08	1.75	1.44	20.41	11.89	4.03	4.58	33.05	26.55	9.07	8.09	35.91	46.79	16.07	24.43
MG13: Vitamin D and other vitamin supplements	8.74	0.95	1.16	2.16	12.94	13.74	3.05	4.28	19.90	27.12	6.70	7.34	24.33	45.95	12.89	22.03
Caplacizumab	0.08	0.00	0.00	NA	0.00	NA	0.00	NA	0.00	NA	0.00	NA	0.00	NA	0.00	NA
Systemic hemostatics	0.00	NA	0.02	0.00	0.03	50.00	0.00	0.00	0.04	50.00	0.00	0.00	0.04	50.00	0.02	15.79
Hereditary angioedema therapeutics	0.00	NA	0.00	NA	0.00	NA	0.00	NA	0.00	NA	0.00	NA	0.00	NA	0.00	NA
Peripheral vasodilators	0.17	0.00	0.05	0.00	0.55	6.06	0.23	0.00	1.13	27.45	0.47	4.43	1.3	38.30	1.10	20.42
Hormonal contraceptives and similar hormone preparations	4.58	0.00	1.31	0.00	3.42	6.80	2.03	0.58	3.13	12.06	2.82	1.88	2.41	30.92	2.81	10.04
Immunoglobulins	0.17	0.00	0.00	NA	0.07	0.00	0.01	33.33	0.09	25.00	0.01	0.00	0.07	100	0.01	20.00
Interferons and CSF	1.42	5.88	0.41	2.04	2.41	28.97	0.54	14.47	2.89	37.69	0.91	22.86	1.18	49.22	0.84	23.78
MG14: NSAIDs and other anti-inflammatory drugs	26.31	1.58	7.31	0.11	36.54	6.96	13.2	1.72	34.5	17.19	18.06	4.15	24.80	35.01	20.18	13.81
MG15: Gout medications	0.67	12.50	0.04	0.00	2.92	15.91	0.63	3.49	6.86	34.95	1.72	10.03	8.39	49.89	3.74	26.46
MG16: Antiepileptics	6.16	6.76	0.71	1.18	9.29	17.35	1.92	7.04	14.5	34.30	3.58	12.07	15.97	47.89	6.89	23.83
MG17: Antipsychotics/Antidepressive drugs	14.32	3.49	3.41	1.47	24.16	12.59	6.92	3.40	36.98	27.63	12.63	7.74	55.58	48.89	27.56	25.11
Rhinological and throat antiseptics	10.49	1.59	3.13	0.27	10.00	5.48	2.92	1.80	7.89	21.97	3.42	3.29	3.95	34.97	3.40	13.93
MG18: Inhaled anti-obstructive drugs	10.49	2.38	2.08	0.40	17.98	9.70	4.22	3.41	25.43	26.38	7.38	8.66	19.44	45.61	9.49	22.23
MG19: Inhaled steroids	2.75	0.00	0.62	0.00	3.62	7.34	0.98	3.63	4.40	21.21	1.50	5.97	2.56	41.37	1.60	16.87
Other COPD drugs	0.92	0.00	0.26	0.00	1.96	5.93	0.33	3.63	1.84	22.89	0.41	7.22	0.88	38.95	0.39	16.04
Cold and cough preparations	7.24	2.30	0.93	0.90	9.27	11.47	1.43	5.34	13.24	26.01	3.06	9.30	12.65	44.97	5.62	26.93
MG20: Systemic antihistamines	5.5	3.03	1.25	0.00	6.03	10.47	1.50	3.05	6.60	22.56	2.34	5.88	6.92	46.07	3.77	23.37
H2 Blockers	0.58	0.00	0.25	0.00	1.3	12.82	0.46	1.11	1.60	22.22	0.84	7.69	1.12	38.84	1.09	17.90

*The medication groups (MG1 to MG20) that were used for further statistical analyses are marked in bold. Detailed numbers are shown in Supplementary Tables S4–S7. It should be noted that NA “not available” means that the calculation was not possible because no patient/control received medication from this group.

Interestingly, we observed that 24.2% of patients aged 41–64 years, 37% of patients aged 65–74 years, and 55.6% of patients aged ≥75 years received antipsychotic/antidepressant drugs before COVID-19 hospitalization. In the control group, the rate of prescribed antipsychotic/antidepressant drugs was 6.9% for patients aged 41–64 years, 12.6% for patients aged 65–74 years, and 27.6% for patients aged ≥75 years.

### 3.2 All-cause mortality for COVID-19 patients

In the 19–40 age group, 1.05% of men and 0.37% of women died in the hospital. One-year mortality rates in this age group were 1.8% for men and 0.93% for women. In older age groups, higher mortality rates were observed. In the 41–64 age group, 5.15% of men and 4.31% of women died during hospitalization. The one-year mortality rate in this age group was 7.91% for men and 6.49% for women. In the 65–74 age group, 17.66% of men and 9.97% of women died during the hospitalization. Within 1 year of hospital admission, 25.38% of men and 14.96% of women died. Furthermore, 33.56% of men and 25.61% of women aged ≥75 years died during the hospitalization. The one-year mortality rate in this age group was 46.2% for men and 39.94% for women (Supplementary Table S8).

In all age groups, we found a trend of a higher risk of death for men than women, which was significant in the older age groups (age >65 years) after multiplicity correction (Supplementary Table S10). The number of hospital admissions per day and the cumulative number of deaths are shown in Figure 1A. The one-year mortality rates are presented in Figure 1B.

**Figure 2:**
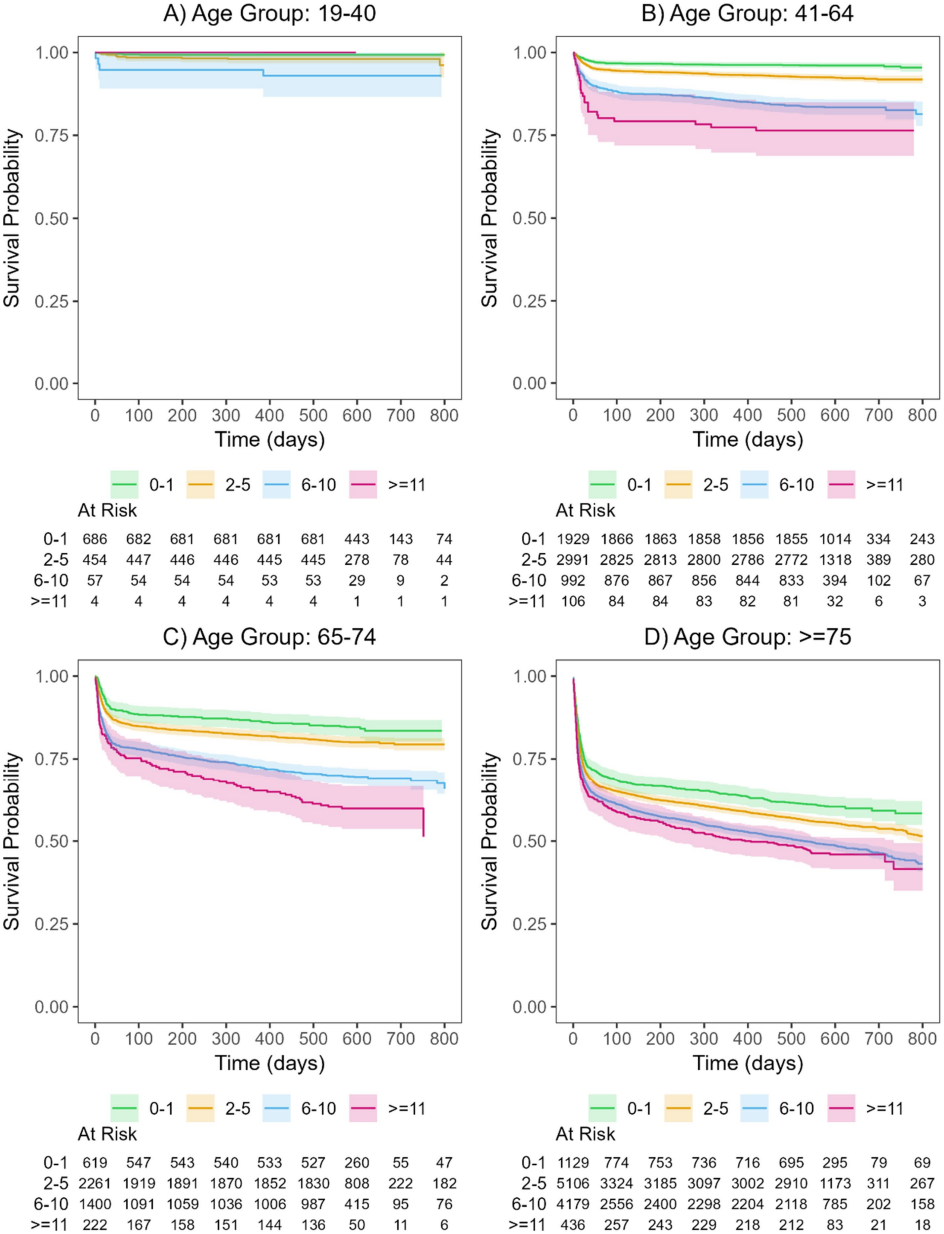
Kaplan-Meier curves and their corresponding 95% confidence intervals for the polypharmacy groups of patients hospitalized with COVID-19 for the four age groups (**A**: 19-40, **B**: 41-64, **C**: 65-74, **D**: >75). For details on hazard ratios, confidence intervals, and p-values see Supplementary Table S10. The curves are shown separately for patients with drugs in 0-1 medication group (green), 2-5 medication groups (orange), 6-10 medication groups (blue), and >=11 medication groups (magenta).

A significantly higher risk of all-cause death was evaluated for patients with a larger number of prescribed medication groups compared to patients receiving drugs from none or only one of the investigated medication groups. No significant relationship was observed for patients aged 19–41 years (Figures 2A–D and Supplementary Figure S1).

Due to the large number of evaluated medication groups and their potential interactions, polypharmacy may represent only one factor in the complex system used to evaluate the associations between medication load and underlying diseases with all-cause death after a hospitalization with COVID-19. Therefore, we additionally included all 20 medication groups in the Cox regression models. Because of the small number of events in the youngest age group (19–40 years), not all 20 medication groups could be included in the model for this age group.

**Figure 3:**
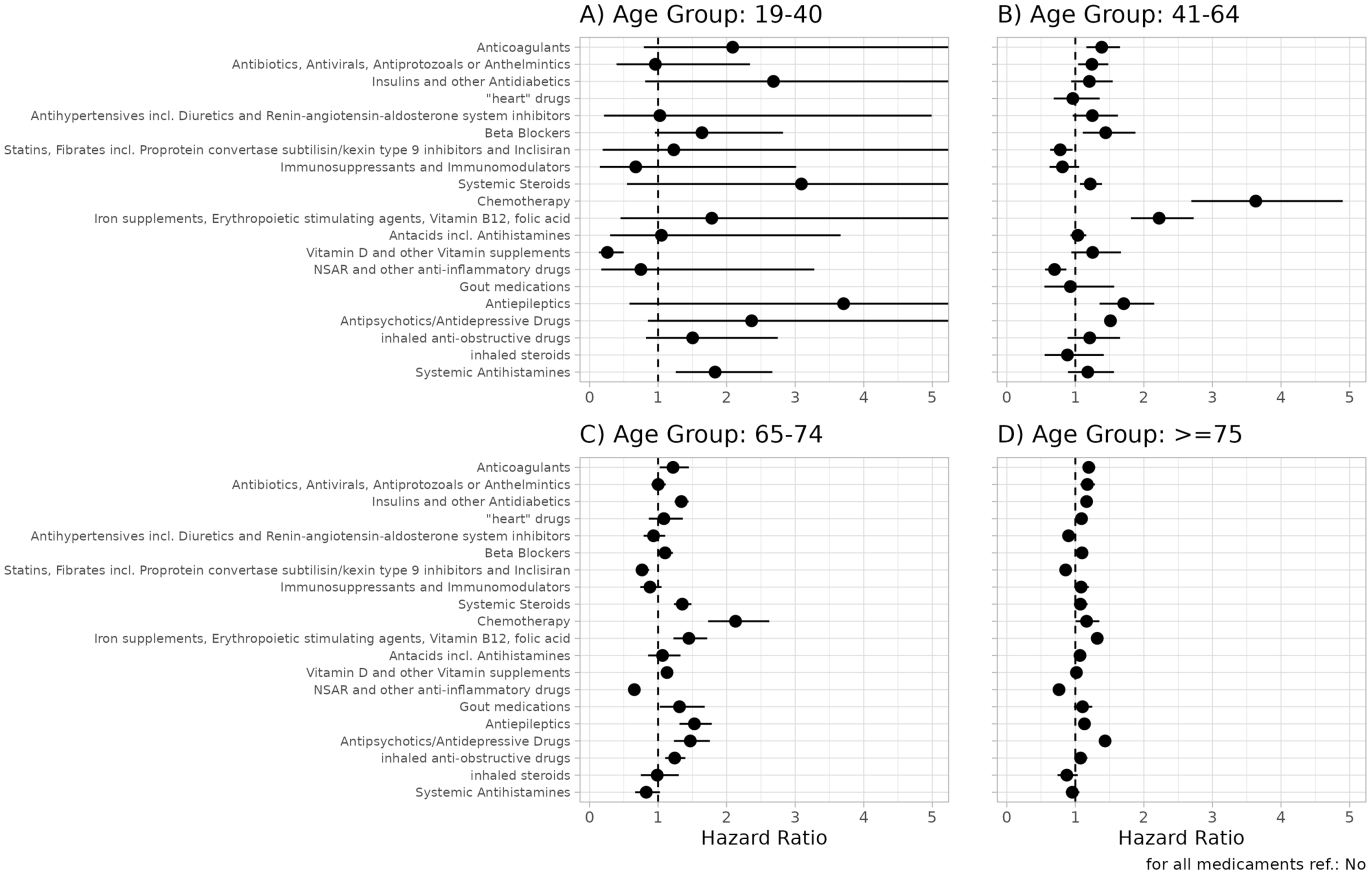
Hazard ratios and 95% confidence intervals for medication groups for the outcome all-cause-mortality in patients hospitalized with COVID-19 for the four age groups (**A**: 19-40, **B**: 41-64, **C**: 65-74, **D**: >75). For details, see Supplementary Table S11. A hazard ratio >1 indicates a higher risk of all-cause death for patients receiving a drug in the corresponding medication group.

Figures 3AD summarizes the results of the Cox regression model of all-cause mortality when including the 20 medication groups (Supplementary Table S11). In the youngest group (19–40 years), only prescribed vitamin D supplements and other vitamins (*p* < 0.001) and systemic antihistamines (*p* = 0.002) were significantly associated with survival. For patients receiving vitamin supplements in the year before hospitalization due to COVID-19, we observed a significantly lower risk of death. In addition, systemic antihistamines were significantly associated with poor survival. The other medication groups did not show significant results. However, due to the small number of events in this age group, these results should be interpreted with caution.

For several medication groups, the results were significantly different between the age groups. Anticoagulants (*p* < 0.001 for 41–64 years and ≥75 years); antibiotics, antivirals, antiprotozoals, or anthelmintics (*p* < 0.001 for ≥75 years); insulin and other antidiabetics (*p* < 0.001 for age groups ≥65 years); heart drugs (*p* = 0.001 for ≥75 years); beta-blockers (*p* < 0.001 for ≥75 years); systemic steroids (*p* < 0.001 for 65–74 years); chemotherapy (*p* < 0.001 for 41–74 years); antacids (*p* < 0.001 for ≥75 years); vitamin D or other vitamin supplements (*p* = 0.001 for 64–74 years); and inhaled anti-obstructive drugs (*p* < 0.001 for 64–74 years) were significantly associated with poor survival. Statins and fibrates were significantly associated with a lower risk of death in patients aged >64 years.

**Figure 4:**
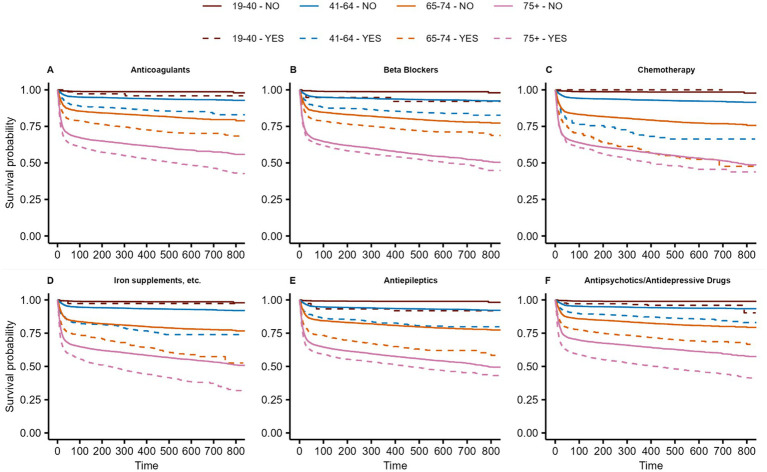
Kaplan-Meier curves of Anticoagulants **(A)**, Beta Blockers **(B)**, Chemotherapy **(C)**, Iron supplements, etc. **(D)**, Antiepileptics **(E)** and Antipsychotics/Antidepressive Drugs associated with poor survival among patients hospitalized with COVID-19. Curves are given for the 4 age groups (brown: 19–40, blue: 41–64, orange: 65–74, magenta: >75) as well for patients with (dashed lines) and without (solid line) the prescribed medication groups. For details on hazard ratios, confidence intervals, and *p*-values, see Supplementary Table S11.

In the three age groups >40 years, a significant association was observed between prescribed antiepileptics (all *p* < 0.001), antipsychotics/antidepressants (all *p* < 0.001), and iron supplements, erythropoiesis-stimulating agents (ESAs), vitamin B12 (B12), and folic acid (FA) (all *p* < 0.001) and a higher risk of all-cause death. For patients receiving non-steroidal anti-inflammatory drugs (NSAIDs) and other anti-inflammatory drugs in the year before hospitalization due to COVID-19, a significantly larger probability for survival was observed (all age groups>40: *p* < 0.001). Figures 4AF presents the Kaplan–Meier curves for key prescribed medication groups associated with poor survival, while Supplementary Figure S6 shows the Kaplan–Meier curves for the medication groups associated with poor survival in the control cohort.

In models that included all investigated medication groups, the polypharmacy variable was not significant, indicating that polypharmacy alone may not be a good predictor of all-cause death. Specific medication groups representing patients with certain underlying diseases may be more critical factors in determining outcomes for hospitalized patients with COVID-19.

### 3.3 All-cause death after COVID-19 hospital survival

Among patients who survived a hospitalization for COVID-19, 0.76% of men and 0.56% of women aged 19–40 years died within the first year after hospital discharge. The 1-year mortality rates were 2.91 and 2.28% for men and women aged 41–64 years, 9.37 and 5.78% for men and women aged 65–74 years, and 19.02 and 19.26% for men and women aged ≥75 years, respectively (Supplementary Table S9).

In the age groups >40 years, we found significant associations with a higher risk of death after surviving a hospitalization for COVID-19 among patients receiving chemotherapy (all *p*-values <0.001) and among those receiving iron supplements, erythropoiesis-stimulating agents, B12, and FA (all *p*-values <0,001). For patients receiving antiepileptics, a significantly higher risk of all-cause death was found in the 41–64 and 65–74 age groups, with an observed trend in the oldest age group (*p* = 0.016). Moreover, patients receiving antipsychotics/antidepressants in the year before hospitalization were significantly associated with poor survival in all age groups >40 years (all *p*-values <0.001).

For NSAIDs and other anti-inflammatory drugs, a significant association with a lower risk of all-cause death after hospital survival was observed in the age group >40 years (*p*-value of <0.001) and for statins and fibrates in the two age groups >65 years (both *p*-values <0.001). Detailed results are shown in Supplementary Table S12 and Supplementary Figure S2.

### 3.4 Re-hospitalization after COVID-19 hospital survival

In the age groups >40 years, a significantly higher risk of readmission was observed due to any cause for patients receiving anticoagulants (all *p* < 0.001), antiepileptics (all *p* < 0.001), systemic steroids (all *p* < 0.001), and chemotherapy (all *p* < 0.008).

We observed a trend toward a higher risk of re-hospitalization for patients receiving antipsychotics/antidepressants (*p* < 0.003 for 19–64 years and ≥75 years; *p* = 0.006 for 65–74 years). Detailed results are shown in Supplementary Table S13 and Supplementary Figure S3.

### 3.5 Comparison of outcomes between COVID-19 patients and controls

The age-, sex-, and region-matched control population received fewer medications than the patient population before hospitalization. Interestingly, in controls without the observed medication groups, remarkably good survival was observed even in the older age groups (Supplementary Figures S4,S5), whereas a steep decrease in survival after hospital admission was observed in patients with COVID-19 (Supplementary Figure S1).

The difference in all-cause mortality between hospitalized patients with COVID-19 and the Austrian control population could not be evaluated in the 19–40 age group due to the small number of events.

For all other subgroups, a significant difference in the risk of all-cause mortality was found between COVID-19-hospitalized patients and PSM controls (all *p* < 0.001). For the subgroup of patients surviving the COVID-19 hospitalization, reduced hazard ratios were observed compared to the PSM controls. The difference between patients and controls remained significant in the age groups 41–64 years (*p* < 0.001) and 65–74 years (*p* < 0.001), but not in the oldest age group (*p* = 0.078). The Kaplan–Meier curves for propensity score-matched COVID-19 patients and controls are shown in Supplementary Figure S7.

Regarding hospitalization for any reason, in all four age groups, a significantly higher probability of re-hospitalization was observed among COVID-19-hospitalized patients compared to controls (all *p* < 0.001). These results suggest that polypharmacy may not completely explain the adverse outcomes in patients with severe COVID-19 (Supplementary Table S14 and Supplementary Figure S3).

## 4 Discussion

In this retrospective study, we evaluated whether baseline medication profiles, which may be interpreted as an indicator for underlying diseases, are associated with survival or hospitalization for any reason after a COVID-19-related hospitalization in an Austrian population. Hospitalized patients with a diagnosis of COVID-19 had a higher medication load before hospitalization and increased long-term mortality, especially patients aged >75 years. Pre-COVID-19 prescriptions of antipsychotic/antidepressant drugs, antiepileptic drugs, chemotherapy, iron/FA/B12, beta-blockers, and anticoagulants were significantly associated with increased mortality, whereas patients who had been prescribed NSAIDs and other anti-inflammatory drugs before hospitalization due to COVID-19 had a significantly lower risk of all-cause death. Due to our study design, we were able to present the “real world” prescription and mortality rate of patients hospitalized with the diagnosis of COVID-19 in Austria, and in a matched control population that was followed from 2020 for up to a maximum of 880 days.

Polypharmacy frequently occurs in patients with COVID-19 and may be associated with increased mortality (15, 16). Analyzing the prescription data of the control population, we detected no drug prescription of investigated medications in 77% (19–40 years), 69% (41–64 years), 56% (65–74 years), and 41% (≥75 years) of controls. In contrast, 30% (19–40 years), 15% (41–64 years), 6% (65–74 years), and 4% (≥75 years) of COVID-19-hospitalized patients had no drug prescriptions.

We found that patients who had been prescribed antipsychotic/antidepressant drugs 1 year before hospitalization had a significantly increased risk of death. Among patients aged ≥75 years in the medication group, the estimated mortality for patients with prescribed antipsychotic/antidepressant drugs was 56.3% (CI: 54.7–57.9) within a 2-year follow-up period as compared to 40.83% (CI: 42.5–39.1) for patients not receiving antipsychotic/antidepressant drugs. Among patients aged ≥75 years in the control group, the mortality rate with prescribed antipsychotic/antidepressant drugs was 32.9% (CI: 32.1–33.7) within a 2-year follow-up as compared to patients not receiving these drugs (11.4%, CI: 11.7–11.1).

The association between anti-inflammatory medications, such as NSAIDs, and lower mortality could be attributed to the pathophysiology of COVID-19, which involves a high pro-inflammatory state in the second phase of the disease, also referred to as the cytokine storm phase (19, 20), which could be attributed to anti-inflammatory medications. In patients with a high pro-inflammatory state in need of oxygen therapy and SARS-CoV-2-associated lung infiltration, anti-inflammatory therapy (e.g., dexamethasone) was the only pharmacological intervention to reduce mortality (21). However, we cannot exclude the possibility of indication bias with patients using NSAIDs regularly potentially being healthier, since patients with more comorbidities (e.g., chronic kidney disease, diabetes, cardiovascular disease, or heart failure) are regularly advised to avoid NSAIDs.

The pronounced association of antipsychotic medication with higher mortality is likely related to the comorbidities present in a population with high prescription rates of such medication, especially at older ages. Antipsychotic/antidepressant drugs are associated with severe COVID-19 morbidity and mortality (16). Antipsychotic medication is primarily prescribed to patients in residential or nursing homes with dementia or behavioral disorders (22, 23). The number of antipsychotic prescriptions is higher in nursing homes (57.1%) than in residential homes (29.5%) (24), emphasizing more frequent prescriptions in a more morbid population. Therefore, chronic use of antipsychotic medications at older ages is most likely indicative of patients with severe impairments (25–27). Antipsychotics are also associated with impaired swallowing, drug-induced dysphagia, and choking, which can lead to detrimental outcomes (28).

Among nursing home residents, a significant positive association was observed between the total number of medications and 30-day, COVID-19-related, adjusted mortality (17). After additional adjustment for dementia and the use of proton pump inhibitors (PPI), vitamin D, antipsychotics/antidepressants, and antithrombotics, this effect was no longer significant, suggesting that polypharmacy itself may not be the issue; rather, the type of medication may be. In our analyses, polypharmacy did not remain significant after correcting for several medication groups.

In patients who were discharged alive after a COVID-19-related hospitalization, the risk of post-discharge death within 180 days among patients aged >64 years was nearly twice as high as that observed in historical controls admitted to the hospital with influenza (12). Furthermore, crude differences in drug use between COVID-19 patients and the general population were identified, particularly in the use of antithrombotic agents, antiepileptics, anti-gout preparations, and cardiac therapy (14).

Scant data are available on clinical outcomes in patients discharged alive from COVID-19 hospitalization. Data from a large study with patients discharged after COVID-19 reported an increased risk of readmission and mortality during a follow-up of 140 days (10). In a German cohort of hospitalized COVID-19 patients, 30-day all-cause mortality was 23.9%, and 180-day all-cause mortality was 29.6%. Another study, after COVID-19 hospitalization of patients in Italy, reported an 8% age-related overall relative increase in all-cause death after 6 months of follow-up (13). Another report investigated the 12-month mortality following recovery from an initial episode of COVID-19 and found a significantly higher adjusted all-cause mortality risk over a 12-month period in patients with severe COVID-19 compared to both COVID-19-negative patients and mild COVID-19 patients. A large study indicated that the overall 2-year mortality risk was worse by day 180 among those infected with COVID-19 compared to matched uninfected comparators (29). In our study, we observed increased long-term mortality and an increased risk of hospitalization for any reason following surviving a COVID-19 hospitalization. Moreover, mortality was more pronounced within the first 50 days after index hospitalization.

The main strength of this study is its use of a large, representative, real-world national database. The retrospective design, however, is a limitation, which we sought to mitigate by including several potential confounding factors in the statistical models and performing propensity score matching to support meaningful comparisons. However, despite the large sample size and long-term follow-up, unmeasured confounding and bias are still possible. The study population was drawn exclusively from the Austrian Health Insurance Fund in 2020, raising potential concerns about the generalizability of these findings to broader patient populations or to later COVID-19 variants. The information used in this study is based on billing records and ATC codes that describe medication load, representing certain underlying comorbidities. However, the contribution of the medication itself or a combination of medications cannot be completely ruled out. Furthermore, we rely on accurate coding across the country, which we cannot verify or correct retrospectively. No information was available from the Austrian Insurance Fund on causes of death or use of intensive care in hospitals.

In conclusion, this large cohort of hospitalized patients with COVID-19 showed an increased short-and long-term risk of mortality. Patients had a higher medication load (polypharmacy). Antipsychotics/antidepressants were significantly associated with poor survival in patients aged >40 years. Our findings may help identify the most vulnerable patients at higher risk of mortality after a COVID-19-related hospital discharge, regardless of age, by screening the prescribed medication groups, with implications for preventive measures. Antipsychotics and antidepressants are assumed to be an underrecognized medication group to identify patients at risk for severe outcomes after hospitalization for COVID-19.

## Data Availability

The datasets presented in this article are not readily available because of data protection. Data that support the findings of this study are available upon reasonable request from the corresponding author. Requests to access the datasets should be directed to Hendrik Jan Ankersmit, hendrik.ankersmit@meduniwien.ac.at.
